# Prediction of massive intraoperative blood transfusion requirement in lung transplantation

**DOI:** 10.3389/fmed.2026.1786557

**Published:** 2026-05-18

**Authors:** Xinchen Tao, Ge Luo, Zhili Xu, Jingcheng Zou, Qi Gao, Yu Yi, Tingting Ni, Junyu Zhou, Qiuping Wang, Yuanyuan Yao, Min Yan

**Affiliations:** Department of Anesthesiology, The Second Affiliated Hospital, Zhejiang University School of Medicine, Hangzhou, China

**Keywords:** lung transplantation, massive transfusion, prediction model, risk factors, transplantation

## Abstract

**Background:**

Massive transfusion (MT) during lung transplantation (LTx) is associated with primary graft dysfunction and increased risk of post-LTx mortality and morbidity. To date, predictive factors for MT during LTx are unknown. This study aimed to establish a predictive model for intraoperative MT in patients undergoing LTx to provide personalized risk prediction.

**Methods:**

This retrospective cohort study included adult patients who underwent LTx at the Second Affiliated Hospital of the Zhejiang University School of Medicine between 2020 and 2023. MT was defined as an intraoperative transfusion of > 5 units of packed red blood cells. The dataset was randomly split into a training cohort (70%) and a test cohort (30%). Three algorithms were applied for variable selection in the training cohort and logistic models were used to establish a predictive model. Model accuracy was assessed using goodness-of-fit tests, receiver operating characteristic analyses, and bootstrap resampling.

**Results:**

In total, 390 patients were included. The MT incidence was 25.9% in the training cohort (*n* = 274) and 25.0% in the test cohort (*n* = 116). The multivariable model for predicting MT incorporated primary diagnosis, hemoglobin level, patient entry route, surgical type, and approach, activated partial thromboplastin time (APTT), and cold ischemia time (CIT). The model demonstrated an AUC of 0.830 (95% CI: 0.775–0.884) in the training cohort and 0.759 (95% CI: 0.657–0.861) in the test set for predicting MT.

**Conclusion:**

A nomogram combining seven predictive factors may help predict the need for intraoperative MT in patients undergoing LTx to optimize individual transfusion strategies.

## Introduction

Lung transplantation (LTx) is a proven treatment for end-stage lung disease that enhances patients’ quality of life and extends survival (1). According to the International Society for Heart and Lung Transplantation (ISHLT) registry, approximately 70,000 adult lung transplants have been recorded, with nearly 4000 performed annually. LTx is associated with higher mortality and complication rates than other solid organ transplants (2, 3), with the potential for significant and unpredictable bleeding. Blood transfusions are the standard treatment for sustained blood loss. Approximately 68% of lung transplant recipients require intraoperative transfusions, with nearly 25% requiring intraoperative massive transfusion (MT) (4). Blood transfusions are linked to increased pulmonary microcirculation pressure, transfusion-related acute lung injury, and mortality, thereby elevating both short- and long-term adverse outcomes (5). Red blood cell (RBC) transfusions heighten mortality and post-transplant complication risks in a dose-dependent manner (6, 7). Multicenter data (*n* = 1,255) revealed that large intraoperative transfusions (> 1 L) are independently associated with primary graft dysfunction (PGD) (6).

Traditional clinical experience and indication-based judgments have limitations in predicting the need for MT in patients with lung transplant and often fail to accurately determine the transfusion needs of individual patients. A clinical prediction model can generate individualized risk assessments and forecast patient outcomes. Accurate assessment of intraoperative MT risk and early identification of high-risk groups are crucial for implementing interventions to minimize transfusion-related complications (8). Additionally, predicting transfusion needs optimizes blood bank resource management by adjusting blood order schedules. Therefore, in this unique population of lung transplant recipients, an effective model to predict the unique risk of intraoperative MT is an urgent need.

Previous studies have examined predictive models for MT in individuals undergoing cardiothoracic surgery and liver transplantation, but these are not generalizable to patients undergoing LTx (9–11). Compared with other surgical populations, patients undergoing LTx exhibit distinct bleeding patterns and transfusion requirements, which are driven by end-stage pulmonary disease, extensive pleural adhesions, pulmonary hypertension, coagulopathy, hemodynamic instability, and the frequent need for extracorporeal support. Therefore, a LTx–specific model is needed for more accurate risk stratification (12, 13). Conversely, in patients undergoing LTx, current research has mainly explored the impact of transfusion on patient prognosis, and no studies have focused on predictors of the need for MT in LTx (4, 14). Therefore, this study aims to develop a predictive model for intraoperative MT in patients undergoing LTx surgery to provide personalized risk prediction and assist management decisions.

## Materials and methods

### Ethics approval

This study was approved by the Ethics Committee of the Second Affiliated Hospital of Zhejiang University School of Medicine, Hangzhou, China (No. 20220352). Due to the retrospective nature of the study, informed consent was waived, and all identifying information was anonymized prior to analysis. This study adhered to the principles of the Declaration of Helsinki.

### Participants

This retrospective study included all adult patients who underwent LTx at the Second Affiliated Hospital of Zhejiang University School of Medicine between 1 January 2020 and 31 December 2023. The following patients were excluded: (1) multi-organ transplantation, including heart-lung transplantation and LTx combined with an extra-thoracic organ transplantation, (2) repeat lung transplantation, (3) data missing rate > 20%. All data were extracted by experienced researchers who were blinded to the details of the study.

### Data collection

Through a literature review, clinical practice, and expert consultations (8, 10, 15), 44 potential predictors were identified using an electronic medical record system, which included demographic data, clinical traits, medical history, laboratory tests, surgical information, and donor information. The demographic data included sex, age, and body mass index (BMI). The preoperative characteristics included primary diagnosis, preoperative comorbidities (previous lung disease, arterial hypertension, pulmonary hypertension, diabetes, pulmonary infection, and cardiac insufficiency), American Society of Anesthesiologists classification (ASA Class), medical conditions, previous surgical history, history of thoracic surgery, preoperative extracorporeal membrane oxygenation (ECMO) use, and preoperative tracheal intubation. Preoperative laboratory tests included hemoglobin, coagulation, complete blood count parameters (including white blood cell count, red blood cell count, hematocrit, and platelet count), albumin, lactate, and serum creatinine levels. Surgical information included type of surgery, surgical access, and patient entry route. Donor information included age, sex, smoking, duration of mechanical ventilation, oxygenation index, cause of death, and cold ischemia time (CIT). For variables assessed multiple times, the measurement closest to the surgery date was selected for the study, and the units of the same indicators were standardized before analysis.

### Clinical practice

LTx was performed via a clamshell incision in the fourth intercostal space, or through a left or right thoracic incision. For bilateral LTx, the less-functional lung was transplanted first. Standard clinical protocols were followed for intraoperative and postoperative care. All patients received general anesthesia via a double-lumen endobronchial tube. Patients with preoperative endotracheal intubation and mechanical ventilation before operating-room arrival were exchanged to a double-lumen tube intraoperatively. During surgery, invasive blood pressure, central venous pressure, pulmonary artery pressure, cardiac index, systemic vascular resistance, and transesophageal echocardiography were monitored. Vasopressors and cardiotonics were administered, as required. The application of ECMO was decided by the surgeon and anesthesiologist. Post-LTx patients were transferred to the intensive care unit for continuous monitoring and management by a multidisciplinary team.

An anesthesiologist managed the intraoperative transfusion of blood products. RBCs were transfused with hemoglobin (Hb) < 8.0 g/dL. Higher transfusion triggers were evaluated based on the patient’s hemodynamic status, blood loss, and surgical complications. Coagulation abnormalities were mainly managed intraoperatively, as clinically indicated, using fresh frozen plasma (FFP), platelets, antifibrinolytics, fibrinogen, and recombinant factor VIIa. Transfusion of blood components was recorded.

### Definition of outcome

Our primary outcome was intraoperative MT, defined as receiving > 5 units of allogeneic RBC transfusion during LTx (4).

### Statistical analysis

Normally distributed data were expressed as mean and standard deviation (SD) and compared using Student’s *t*-test. Non-normally distributed variables are presented as medians with interquartile ranges (IQR) and were analyzed using the Mann–Whitney U test. Categorical variables are expressed as counts and percentages and were analyzed using Pearson’s chi-square test or Fisher’s exact test, depending on suitability. When the missing data was below 10%, multiple imputation was employed to fill in the gaps.

The data were divided randomly into a training cohort (70%) for model development and a test cohort (30%) for evaluation. To ensure the randomization was reliable, the two cohorts were compared using baseline recipient, donor, and surgical variables. The prediction model was created with the training cohort and then tested on the test cohort.

### Variable selection

In the development cohort, we employed three methods to screen for predictor variables, and predictors with a *P*-value < 0.10 from univariate analyses were selected using a backward stepwise multivariate logistic regression model guided by the minimum Akaike information criterion (AIC). Second, the least absolute shrinkage and selection operator (LASSO) regularization algorithm with a 10-fold cross-validation was employed to identify potential predictors with non-zero coefficients. Third, a streamlined model was developed using the Random Forest Recursive Feature Elimination (RF-RFE) method combined with backward stepwise selection. In the training cohort, the variable selection performance was evaluated using Nagelkerke R (higher values indicating better performance), Root Mean Square Error (RMSE, where lower values are preferable), and Bayesian Information Criterion (BIC, with lower values being more favorable).

Ultimately, the best feature selection method was identified by applying Occam’s razor principle while evaluating the model performance. Non-linear continuous variables were converted into categorical variables using the restricted cubic spline (RCS) function to check the linearity between the identified continuous variables in the model and the log odds outcome. Collinearity among predictors was evaluated using the variance inflation factor (VIF), where a VIF of ≥ 5 suggested multicollinearity (16).

### Development and validation of the prediction model

Multivariate logistic regression analysis was used to develop the predictive models. Validation of the model in the test cohort involved using the Hosmer-Lemeshow goodness-of-fit test for calibration assessment and the area under the receiver operating characteristic curve (AUC) for evaluating discriminative power. Bootstrap calculations of the AUC were obtained using 1,000 resamples for validation. Calibration curves were used to evaluate the agreement between observed and predicted outcomes. An ideal model offers accurate predictions, with calibration curves matching the diagonal. The performance of the nomogram improved as its calibration curve approached the diagonal, and bias-corrected calibration curves were demonstrated using 1,000 bootstrap resamples. Model calibration was further quantified using the Brier score, with values > 0.30 denoting suboptimal calibration. The H-L goodness-of-fit test *p*-value > 0.05 indicated no significant difference between the observed and predicted values. Decision curve analysis (DCA) was used to assess the model’s net benefit (NB) for clinical applicability by balancing true positives with false negatives. Finally, we translated the multivariate logistic regression into a nomogram. Regression coefficients (β) were rescaled to a 0–100 point scale, and the sum of these points corresponded to each patient’s estimated probability of intraoperative MT.

In the exploratory analysis, we assessed blood-product utilization under a scenario in which model predictions guided preoperative ordering. Overuse of blood bank resources was defined as the absolute difference between the number of preoperatively ordered units of packed RBC (pRBC) and the number of units actually transfused (10). After selecting a probability threshold via DCA, we partitioned patients into predicted MT and predicted non-MT groups and compared three ordering strategies in the test cohort. First, we computed overuse directly from the observed (actually placed) preoperative orders. Second, we posited a fixed-tier ordering protocol that assigned 10 units to patients predicted to require intraoperative MT and 5 units to those predicted not to require intraoperative MT (bundles routinely used by our institution’s blood bank). Finally, for patients in each predicted group, we determined the optimal pRBC order quantity to evaluate overuse. By applying a quadratic penalty function to the deviation between actual units transfused and units ordered preoperatively, the optimal bundle size at a given threshold equaled the within-group mean of actual units transfused for each of the two groups.

All analyses were conducted using R v.4.2.2 (R Foundation for Statistical Computing).^1^ The R packages “rms,” “pROC,” “MASS,” “survival,” and “dcurves” were used, with a two-sided *P* < 0.05 deemed statistically significant.

## Results

### Patient characteristics

We screened 399 adult patients who underwent LTx. Nine patients were excluded, including five with multi-organ transplants and four with secondary transplants. Ultimately, 390 patients were included in the analysis (Supplementary Figure 1). Supplementary Figure 2 shows the variables with missing patient data. Missing data were limited to donor-related variables.

In our cohort, the patients had an average age of 56.9 ± 11.7 years. Patients were male (83.3%). The most common cause of lung disease was interstitial lung disease (ILD) (47.4%), followed by chronic obstructive pulmonary disease (COPD) (21.5%) (Table 1). Intraoperatively, the patients received a median (IQR) of 1.5 (0–5) units of packed RBCs and 405 (0–955) mL of FFP. Of the 390 patients, 27 (6.9%) received platelet transfusions and six (1.5%) received cold precipitation. Seventy (17.9%) patients received recovered autologous blood. The training and test cohorts included 274 and 116 patients, respectively. Table 1 shows the baseline characteristics of the patients in both the training and test cohorts, with no significant differences between them.

**TABLE 1:** Baseline characteristics of patients in the training and test cohorts.

Characteristic	Training cohort	Test cohort	*P*-value
	(*n* = 274)	(*n* = 116)	
Baseline characteristics			
Age(years), mean (SD)	57.3 (11.1)	55.8 (12.9)	0.264
Sex, n (%)			0.347
Male	232 (84.7%)	93 (80.2%)	
Female	42 (15.3%)	23 (19.8%)	
BMI (kg/m^2^), mean (SD)	21.0 (4.07)	20.6 (4.13)	0.422
Comorbidities and medical history, n (%)			
COPD	80 (29.2%)	37 (31.9%)	0.681
Pulmonary infection	110 (40.1%)	46 (39.7%)	> 0.999
Pleural effusion	36 (13.1%)	9 (7.76%)	0.178
Respiratory failure	199 (72.6%)	89 (76.7%)	0.474
Coronary artery disease	54 (19.7%)	28 (24.1%)	0.398
Hypertension	36 (13.1%)	18 (15.5%)	0.645
Heart dysfunction	41 (15.0%)	14 (12.1%)	0.554
Pulmonary hypertension	143 (52.2%)	71 (61.2%)	0.127
Cerebral infarction	7 (2.55%)	1 (0.86%)	0.445
Diabetes	41 (15.0%)	10 (8.62%)	0.125
Surgery-related characteristics, n (%)			
Primary Diagnosis			0.977
COPD	58 (21.2%)	26 (22.4%)	
ILD	132 (48.2%)	53 (45.7%)	
Pneumoconiosis	41 (15.0%)	18 (15.5%)	
Others	43 (15.7%)	19 (16.4%)	
ASA classification			0.885
III	30 (10.9%)	14 (12.1%)	
IV-V	244 (89.1%)	102 (87.9%)	
Surgical type			0.339
Unilateral LTx	103 (37.6%)	37 (31.9%)	
Bilateral LTx	171 (62.4%)	79 (68.1%)	
Surgical approach			0.109
Anterior external	213 (77.7%)	94 (81.0%)	
Posterior external	26 (9.49%)	4 (3.45%)	
Clamshell	35 (12.8%)	18 (15.5%)	
Previous surgical history	81 (29.6%)	35 (30.2%)	>0.999
History of thoracic surgery	10 (3.65%)	3 (2.59%)	0.763
Preoperative ECG abnormalities			0.372
None	231 (84.3%)	95 (81.9%)	
Supraventricular	17 (6.20%)	5 (4.31%)	
Ventricular	26 (9.49%)	16 (13.8%)	
Preoperative tracheal intubation	70 (25.5%)	23 (19.8%)	0.279
Preoperative ECMO	55 (20.1%)	20 (17.2%)	0.611
Patient entry route			0.121
Ward	177 (64.6%)	85 (73.3%)	
ICU	97 (35.4%)	31 (26.7%)	
Intraoperative Dialysis	21 (7.66%)	5 (4.31%)	0.321
Intraoperative ECMO			0.419
None	21 (7.66%)	11 (9.48%)	
VV	209 (76.3%)	92 (79.3%)	
VA	44 (16.1%)	13 (11.2%)	
Preoperative laboratory data, mean (SD)			
Hemoglobin(g/L)	126 (24.2)	124 (25.1)	0.466
Platelets(10^9^/L)	194 (78.9)	203 (84.6)	0.337
Creatinine(μmol/L)	64.0 (23.6)	63.7 (23.3)	0.906
GLU(mmol/L)	7.56 (2.76)	7.41 (2.85)	0.647
PT(s)	13.4 (1.46)	13.2 (1.32)	0.198
PTA(%)	100 (18.0)	103 (20.8)	0.167
INR	1.02 (0.15)	1.01 (0.14)	0.390
APTT(s)	37.6 (10.5)	36.8 (9.48)	0.495
TT(s)	20.7 (25.1)	19.2 (20.8)	0.565
FBG(g/L)	3.87 (1.19)	4.00 (1.28)	0.353
DD(ng/mL)	1573 (3399)	1366 (2442)	0.499
PCO_2_(mmHg)	47.1 (13.0)	48.2 (13.4)	0.456
SATO_2_(%)	97.7 (3.69)	97.4 (3.29)	0.498
Donor-related characteristics			
Donor sex, n (%)			0.179
Female	42 (15.3%)	25 (21.6%)	
Male	232 (84.7%)	91 (78.4%)	
Donor age(years), mean (SD)	43.7 (10.6)	43.9 (11.3)	0.918
Donor type, n (%)			>0.999
DBD	265 (96.7%)	112 (96.6%)	
DCD	9 (3.28%)	4 (3.45%)	
Donor ventilator time(days), mean (SD)	11.3 (21.1)	14.2 (32.8)	0.385
Donor PaO_2_/FiO_2_ (mmHg), mean (SD)	407 (87.5)	408 (76.5)	0.927
CIT (hours), mean (SD)	7.16 (1.79)	7.39 (1.51)	0.201

Variables are presented as the n (%) for categorical variables or the mean (SD) for continuous variables. LTx, lung transplantation; BMI, body mass index; COPD, chronic obstructive pulmonary disease; ILD, interstitial lung disease; ECMO, extracorporeal membrane oxygenation; ICU, intensive care unit; VV, veno-venous; VA, veno-arterial; GLU, glucose; PT, prothrombin time; PTA, prothrombin activity; INR, international normalized ratio; APTT, activated partial thromboplastin time; TT, thrombin time; FBG, fibrinogen; DD, D-dimer; PCO2, partial pressure of carbon dioxide; SaO2, arterial oxygen saturation; PaO2/FiO2, ratio of arterial oxygen tension to inspired oxygen fraction; CIT, cold ischemia time; SD, standard deviation.

### Intraoperative massive transfusion

Among the 390 patients, 208 (53.3%) underwent RBC transfusion during LTx surgery, with 100 (25.6%) receiving > 5 units (Supplementary Figure 3). The median number of RBCs infused in the MT group was 8.5 (7.0–13.5) units, with a maximum of 40.5 units. RBC units infused in the MT group accounted for 78.6% of the total RBC units infused (1139/1450 units) in all patients during the study period. The incidence of intraoperative MT was 25.9% in the training cohort and 25.0% in the test cohorts.

### Correlation heatmap of predictor variables in the training cohort

Supplementary Figure 4 displays the correlation matrix for the training cohort, where the color intensity reflects the correlation strength (blue for negative and red for positive); most predictors exhibit only weak correlations.

### Variable selection

**FIGURE 1:**
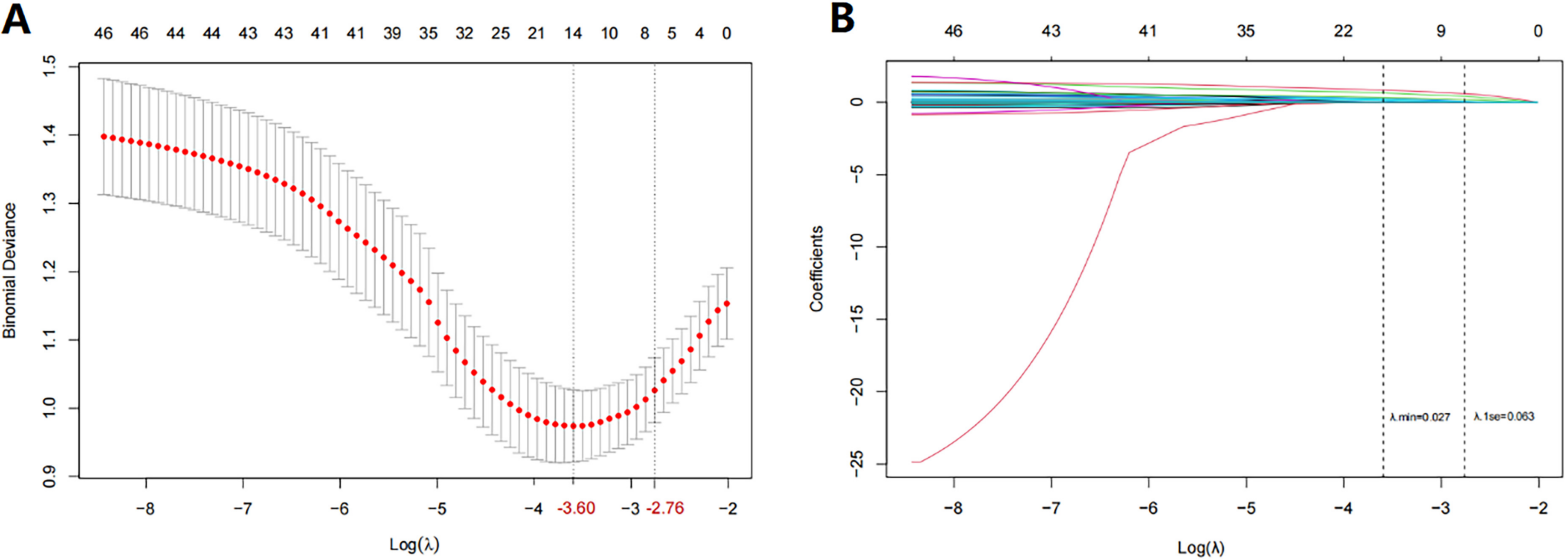
Variable selection using least absolute shrinkage and selection operator (LASSO) regression in the training cohort. **(A)** LASSO-based logistic model with 10-fold cross-validation to determine the optimal parameter (λ) by minimizing the binomial deviance. Two vertical dashed lines mark the λ values chosen according to two criteria: the minimal binomial deviance (λ_*min*_) and one standard error of the minimum (λ_1s*e*_). The log (λ_1s*e*_) of - 2.39 and λ_1s*e*_ of 0.063 were considered optimal. **(B)** A LASSO coefficient profile of all feature variables against the log (λ) sequence is shown. The resulting 7 predictors with non-zero coefficients were identified based on the log (λ_1s*e*_) value.

Univariate and multivariate logistic regression analyses were conducted to evaluate variables associated with intraoperative MT (Supplementary Table 1). Multivariate analysis identified 6 candidate predictors (Supplementary Table 1 and Supplementary Figure 5A). Figure 1A shows the outcomes of the 46 variables incorporated in the LASSO regression. With an increase in λ to 0.063, corresponding to one standard error of the minimum λ, the model retained only 7 candidate predictors, potentially having the most significant impact on distinguishing intraoperative MT (Figure 1B and Supplementary Figure 5B). The RF-RFE method was most accurate when it included 29 predictors (Supplementary Figure 6A); its importance score is shown in Supplementary Figure 6B. Fourteen candidate predictors were identified using backward stepwise regression (Supplementary Figure 5C).

These methods were thoroughly assessed using the training cohort, focusing on AUC, overall accuracy, and model performance indices (Table 2). The LASSO regression model was determined to be the best-performing basic model, with seven predictors: primary diagnosis, hemoglobin (Hb), patient entry route, surgical type, surgical approach, activated partial thromboplastin time (APTT) and CIT. Hb, APTT, and CIT were analyzed using the RCS model, and the non-linear continuous predictor, CIT, was transformed into a categorical predictor (Supplementary Figure 7).

**TABLE 2:** Performance of three feature variable selection in the training cohort.

Feature selection method	No. of feature variables	AUC (95% CI)	Overall accuracy (95% CI)	Nagelkerke *R*^2^	BIC	Brier score
Univariate and multivariate stepwise regression	6	0.824 (0.769, 0.879)	0.818 (0.767, 0.861)	0.37	284.44	0.137
LASSO regression	7	0.830 (0.775, 0.884)	0.810(0.759, 0.855)	0.373	295.02	0.136
RF-RFE and stepwise regression	14	0.851 (0.798, 0.904)	0.825 (0.775, 0.868)	0.447	314.96	0.122

AUC, area under the receiver operating characteristic curve; BIC, Bayesian information criterion; CI, confidence interval; LASSO, least absolute shrinkage and selection operator; RF-RFE, random forest-recursive feature elimination algorithm.

**FIGURE 2:**
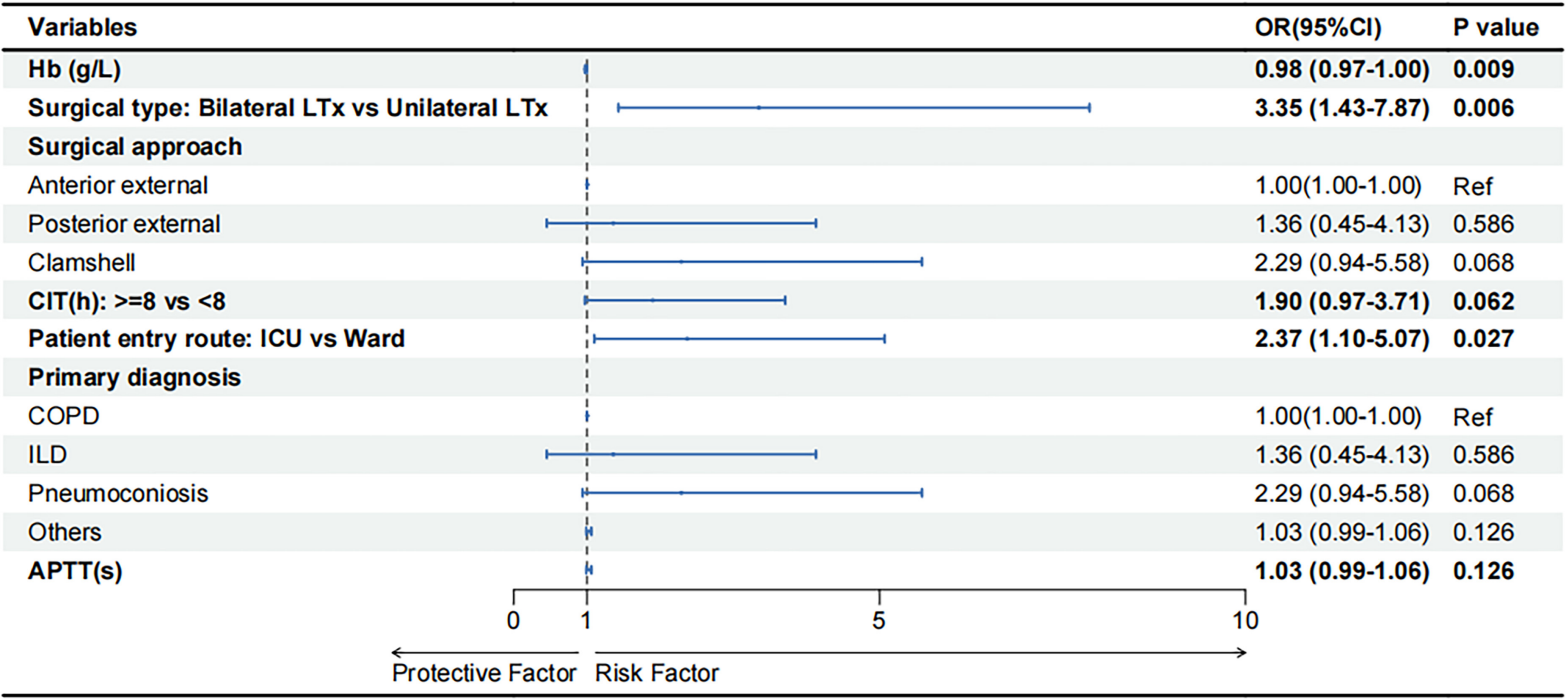
Forest plot for predictors included in the prediction model. Abbreviations: OR, odds ratio; CI, confidence interval; Hb, hemoglobin; LTx, lung transplantation; CIT, cold ischemia time; ICU, intensive care unit; COPD, chronic obstructive pulmonary disease; ILD, interstitial lung disease; ICU, intensive care unit; APTT, activated partial thromboplastin time.

The prediction model’s final predictors comprised primary diagnosis (ILD, COPD, pneumoconiosis, others), Hb (g/L), patient entry route (Ward vs. ICU), surgical type (unilateral LTx vs. bilateral LTx), surgical approach (anterior external, posterior external, clamshell), APTT (s) and CIT (< 8 h vs. ≥ 8 h). Figure 2 shows the OR and 95% CI of the predictors mentioned above.

### Development and validation of the model for predicting MT

**FIGURE 3:**
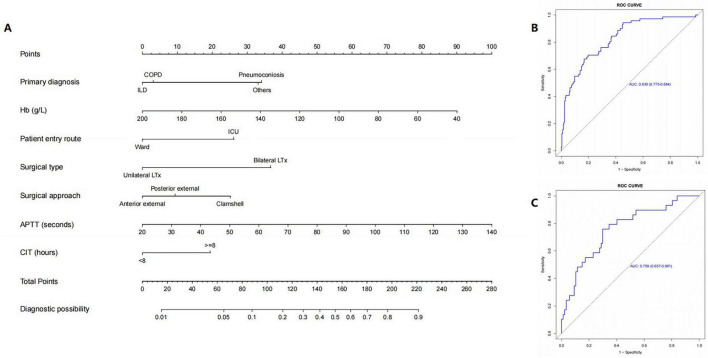
Development and application of a model for predicting the risk of MT during LTx. **(A)** Nomogram for MT during LTx. **(B)** ROC curves showing the performance of the prediction model in predicting MT in the training cohort. **(C)** ROC curves showing the performance of the prediction model in predicting MT in the test cohort. ROC, receiving operating characteristic; MT, massive transfusion; Hb, hemoglobin; LTx, lung transplantation; CIT, cold ischemia time; ICU, intensive care unit; COPD, chronic obstructive pulmonary disease; ILD, interstitial lung disease; ICU, intensive care unit; APTT, activated partial thromboplastin time.

**FIGURE 4:**
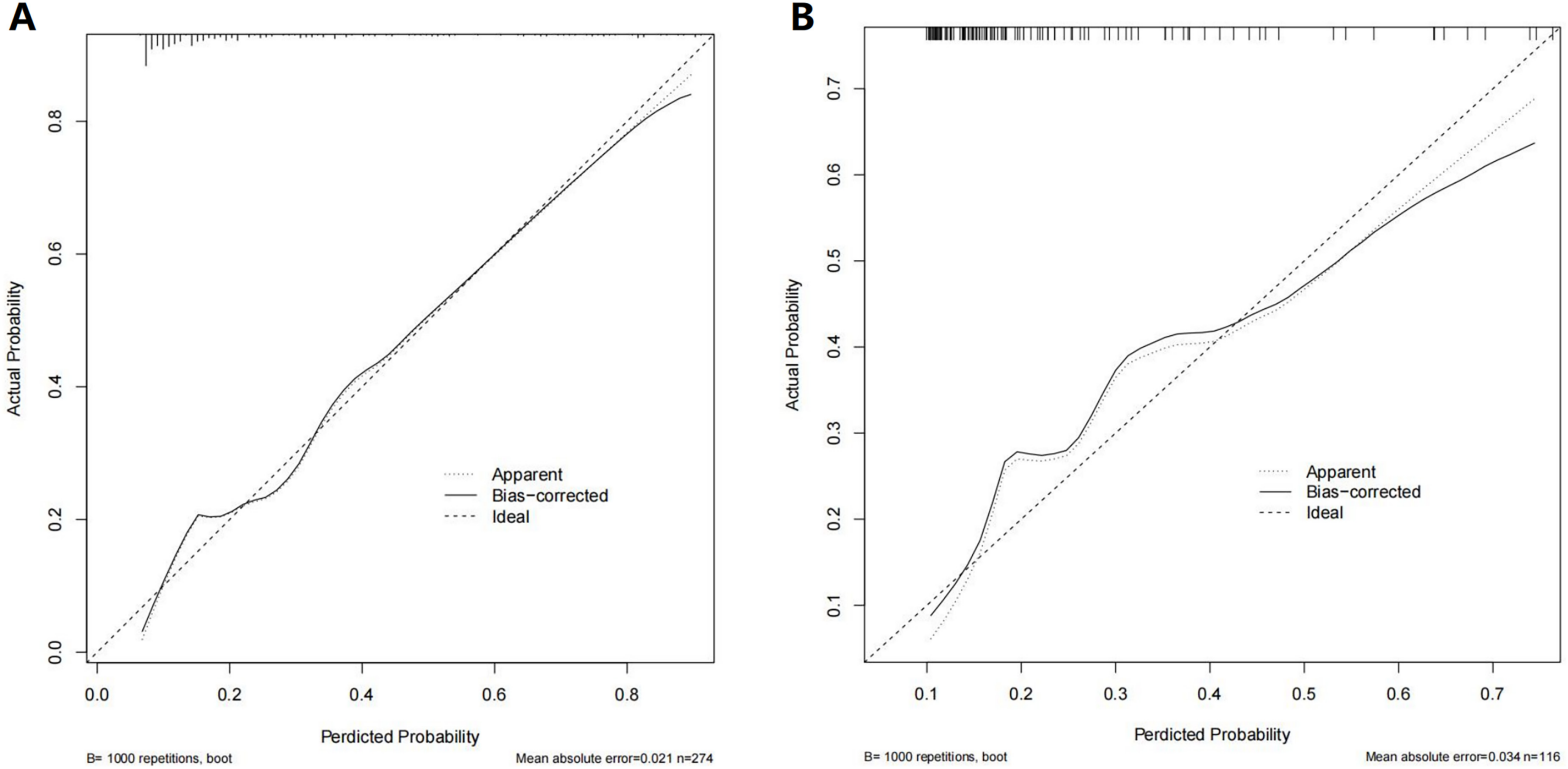
Calibration curves for testing the stability of prediction model in the training and test cohorts. **(A)** Calibration curve for MT in the training cohort. **(B)** Calibration curve for MT in the test cohort. MT, massive transfusion.

Seven predictors were used to create a nomogram model, enabling the calculation of the predicted risk scores for each patient (Figure 3A). In the nomogram, preoperative APTT had the greatest effect on intraoperative MT occurrence, followed by preoperative Hb level, surgical type, primary diagnosis, patient entry route, surgical approach, and CIT. In the training cohort, the AUC was 0.830 (95% CI: 0.775–0.884), with a corrected AUC of 0.846 (95% CI: 0.761–0.866). Calibration using 1000 bootstrap curves demonstrated a strong concordance between the predicted and observed outcomes (Figure 3B). The Brier score was 0.137, and the H-L Chi-square value was 9.700 (P = 0.287) (Figure 4A). The test set yielded an AUC of 0.759 (95% CI: 0.657–0.861) (Figure 3C). The Brier score was 0.159, and the H-L Chi-square value was 11.675 (*P* = 0.166), indicating good model calibration (Figure 4B).

DCA was conducted on both the training and test sets of the predictive model to assess its clinical utility by quantifying the net benefits across various threshold probabilities. In the training cohort, employing the predictive model to guide clinical interventions offered greater benefits compared with scenarios in which all patients were treated (red line) or no patients were treated (green line, Supplementary Figure 8A) when a patient’s MT threshold probability was > 4%. The corresponding threshold probability in the test set was 10% (Supplementary Figure 8B). Among the 116 patients in the test set, using 0.10 as the probability threshold, the 10-unit or 5-unit allocation guided by MT prediction would save 261 units of pRBCs per 100 patients compared with observed ordering, whereas the fully optimized allocation would save 535 units of pRBC per 100 patients.

## Discussion

We constructed a model for predicting intraoperative MT during LTx, incorporating the primary diagnosis, Hb level, patient entry route, surgical type, surgical approach, APTT, and CIT as key predictors. Our MT model showed a satisfactory discriminatory performance in identifying high-risk patients. Internal validation confirmed that the model exhibited good calibration and predictive abilities. The application of such a simple visual model could reduce blood product waste by predicting MT requirements and tailoring transfusion regimens.

This study found a 25.6% incidence of intraoperative MT, slightly exceeding the 18% reported by Atchade et al. in 147 lung transplant patients (4). This difference may stem from variations in patient conditions and transfusion strategies. As LTx indications have broadened, recipients more often present with pulmonary hypertension or coagulopathy, increasing transfusion needs. In addition, Atchade et al. used a stricter threshold (Hb < 7 g/dL) than ours (8 g/dL, with escalation based on clinical status) (4). Notably, Cernak et al. defined MT as transfusion of > 10 units of RBCs within 24 h of surgery, with a reported incidence of 27% (17).

This study aligns with previous research indicating that preoperative Hb levels correlate with the risk of MT in liver transplantation and cardiac surgery (10, 11, 18). A large multicenter prospective observational study identified a low preoperative Hb concentration as the primary reason for intraoperative transfusion (19), likely because of its impact on the anemia status and oxygen delivery capacity of the patient (20). Unlike non-modifiable anatomic and surgical factors, anemia affords a well-defined window for intervention both during the lung-transplant waiting period and throughout the perioperative phase. These findings underscore the importance of managing preoperative anemia to decrease intraoperative blood transfusions in lung transplant recipients.

Bilateral LTx was associated with a 3.08-fold increased risk of MT compared with that of unilateral LTx, due to its technical complexity. Bilateral LTx requires a prolonged operative time, stripping of bilateral thoracic adhesions, and sequential vascular anastomosis, all of which increase the risk of bleeding. These findings align with those of Wang et al. in that patients undergoing double LTx have a greater need for blood products (8). Bilateral LTx recipients require more blood products because of the complexity of the procedure and the increased use of extracorporeal circulation (21).

This study included primary diagnosis as a variable in the nomogram. Wang et al. found that patients with Eisenmenger syndrome (ES) and cystic fibrosis (CF) required more blood products than those with ILD and COPD, aligning with previous research. This pattern may reflect differences in disease-related anatomy, the need for bilateral transplantation or extracorporeal support. Additionally, adhesions due to persistent inflammation make recipient lung removal more challenging than that in patients with COPD, where adhesions are rare. Our findings indicate that pneumoconiosis is linked to an increased risk of MT, potentially due to the intricate pulmonary arteriovenous structure, severe thoracic adhesions, and frequent occurrence of pulmonary hypertension in these patients (22). Wang et al. did not report this phenomenon in their study. The relatively high prevalence of pneumoconiosis among Chinese lung transplant recipients suggests that the population of lung transplant recipients in China has a unique etiology (23, 24).

The nomogram showed that patients transferred from the ICU were associated with a greater risk of intraoperative MT than those entering the operating room from the ward (OR = 2.37, 95%CI 1.10–5.07). Preoperative ICU patients may have more severe systemic pathological conditions, often with decreased coagulation reserve function and systemic inflammatory activation, and more patients receiving mechanical ventilation and ECMO, increasing the risk of intraoperative bleeding (25, 26). This suggests that stratified preoperative management should be implemented for ICU patients to optimize coagulation control.

We found that the surgical approach, APTT, and CIT were important factors influencing intraoperative MT. The limited attention given to these factors in the context of LTx underscores the innovation and significance of our model. This study identified that clamshell incisions posed a greater risk of MT compared with posterolateral incisions, whereas anterolateral incisions were linked to the lowest MT risk. This difference may stem from the anatomical and technical characteristics of the different access routes. The clamshell incision, which provides optimal visualization, involves more extensive mediastinal dissection and pericardial manipulation, increasing the risk of vascular injury and hemodynamic instability. (27) Conversely, the posterolateral incision preserves the integrity of the sternum and reduces exposure of the mediastinal structures, while providing adequate hilar visualization and decreasing the risk of hemorrhage (28). Senbaklavaci et al. reported that the clamshell incision approach involved extensive mediastinal dissection, potentially increasing the risk of lymphatic and small vessel injury, as well as that of intraoperative bleeding (29). Therefore, surgical access should be considered in the MT risk assessment, with an anterolateral incision being preferable when clinically feasible to balance exposure and bleeding risk.

The association between prolonged preoperative APTT and increased risk of MT has an important physiological basis. Lung transplant candidates often present coagulation disorders due to chronic lung disease, abnormal liver function, or anticoagulation therapy (30). Loizou et al. noted that abnormally prolonged preoperative APTT is linked to increased bleeding risk during surgery and anesthesia (31). Although coagulation markers are established predictors of MT in various surgical interventions, their role in lung transplant patients remains unexplored (15, 32). This highlights the importance of preoperative coagulation evaluation and optimization.

Prolonged CIT was associated with a higher risk of intraoperative MT in our study. This association may reflect several factors. Longer ischemic times may be linked to ischemia-reperfusion injury and coagulation disturbances (33). Furthermore, CIT may also serve as a surrogate marker of the overall complexity of donor lung procurement, transport, graft assessment, and transplantation procedures. Therefore, CIT should be interpreted cautiously as a composite perioperative indicator rather than a purely biological risk factor. Okamoto et al. revealed that donor lungs with a CIT > 8 h significantly impaired transplantation suitability and post-transplant function (33). However, further research is required to elucidate effects on transfusion requirements, which highlights the clinical importance of enhancing donor organ procurement efficiency and inter-transplant transfer coordination to minimize CIT.

The nomogram offers a practical tool to enhance clinical decision-making in MT. In our study, the model demonstrated strong performance, achieving an AUC of 0.83 in the training cohort and maintaining good predictive accuracy with an AUC of 0.76 in the test cohort. The calibration curve confirmed the model’s reliability through the close alignment of predicted and actual results. Furthermore, the DCA showed that use of a nomogram to predict MT provided a net benefit for most patients.

Crucially, our study compared three preoperative blood-ordering strategies. Compared with the conventional practice guided by surgeon discretion, a data-driven, risk-stratified strategy may improve the utilization of blood resources and reduced over-ordering. Clinically, the crossmatch-to-transfusion (C/T) ratio is commonly used to judge the appropriateness of RBC orders, with a benchmark of ≤ 2 often regarded as ideal across surgical procedures (34). However, for operations such as LTx where bleeding risk varies substantially, a one-size-fits-all ordering policy may lead to unnecessary waste. Tailoring preoperative orders to each patient’s individualized probability of MT may lower the C/T ratio without increasing the risk of emergency release of uncrossmatched blood, thereby providing a rationale for shifting from experience-based to evidence-based blood ordering.

Currently, no alternative models exist for predicting MT in patients undergoing LTx. This study enables the tailoring of transfusion regimens based on preoperative risk assessment and provides a basis for more individualized and targeted interventions. This model provides a foundation for future research, enabling the exploration of additional predictors or the refinement of the nomogram to reduce intraoperative transfusions and optimize blood-ordering strategies.

This study has several limitations. First, this was a retrospective analysis, and the collected data may have an inherent bias. Second, transfusion strategies in LTx may vary owing to differences in center experience, which may affect the external validity of the results. Additionally, the reliability of the nomogram requires further confirmation through prospective studies. The single-center design necessitates external validation using data from additional centers.

## Conclusion

We developed a novel nomogram to predict intraoperative MT in patients undergoing LTx. This tool allows clinicians to quickly evaluate the risk of intraoperative MT in patients at the preoperative stage, thereby enabling individualized blood management strategies and improving perioperative decision making and resource allocation.

## Data Availability

The raw data supporting the conclusions of this article will be made available by the authors, without undue reservation.

## Footnotes

1
https://www.r-project.org/
