# Supplementary material for: Prediction of massive intraoperative blood transfusion requirement in lung transplantation
Source: Front Med (Lausanne). 2026 May 18;13:1786557. doi: 10.3389/fmed.2026.1786557 (PMC13222940; doi:10.3389/fmed.2026.1786557)

**Supplementary file**

**sFigure 1.** Flowchart for selection of study participants.


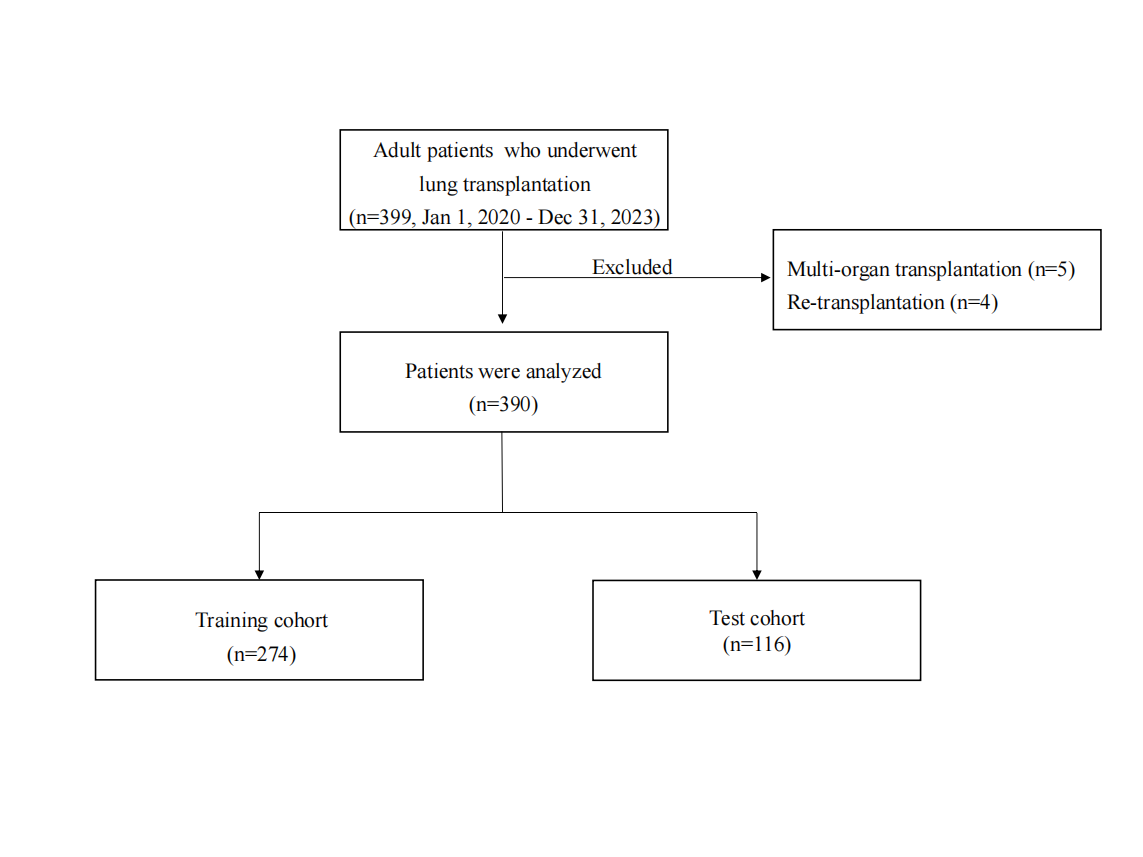


**sFigure 2.** Visual presentation of proportion and pattern of missing data in donor information. Missing proportion of each variable is shown in the upper histogram. In the missing pattern plot, variables with missing or non-missing data were indicated by grey or blue squares respectively with ratio columns of all corresponding missing patterns on the bottom. Abbreviations in this figure are the same as those described previously. Note: Only donor information is missing from our dataset.


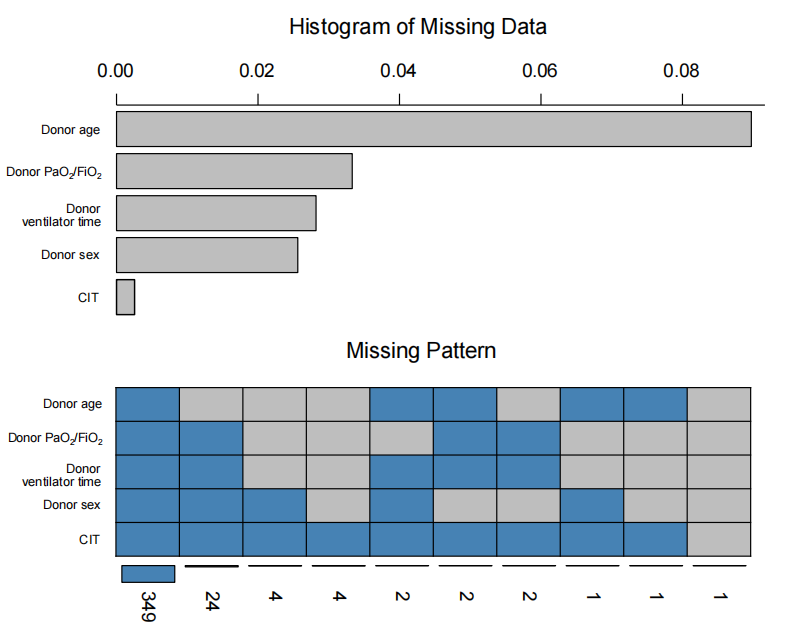


**sFigure 3.** The number of patients on different RBC transfusion volumes.
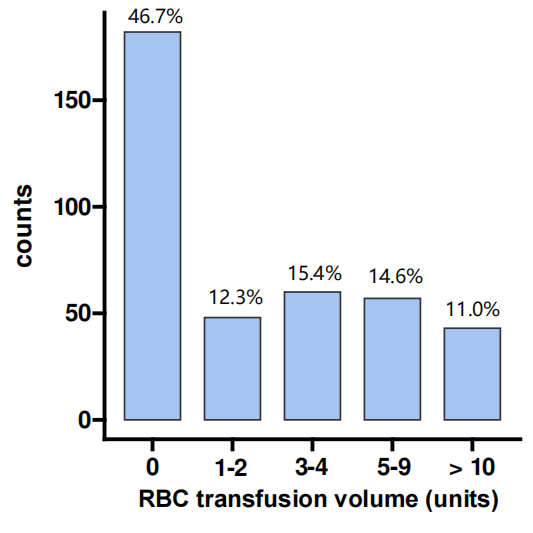


**sFigure 4.** Correlations between variables. The correlation is reflected by the color intensity in the scale (blue=negative correlation, red=positive correlation). Abbreviations in this figure are the same as those described previously.


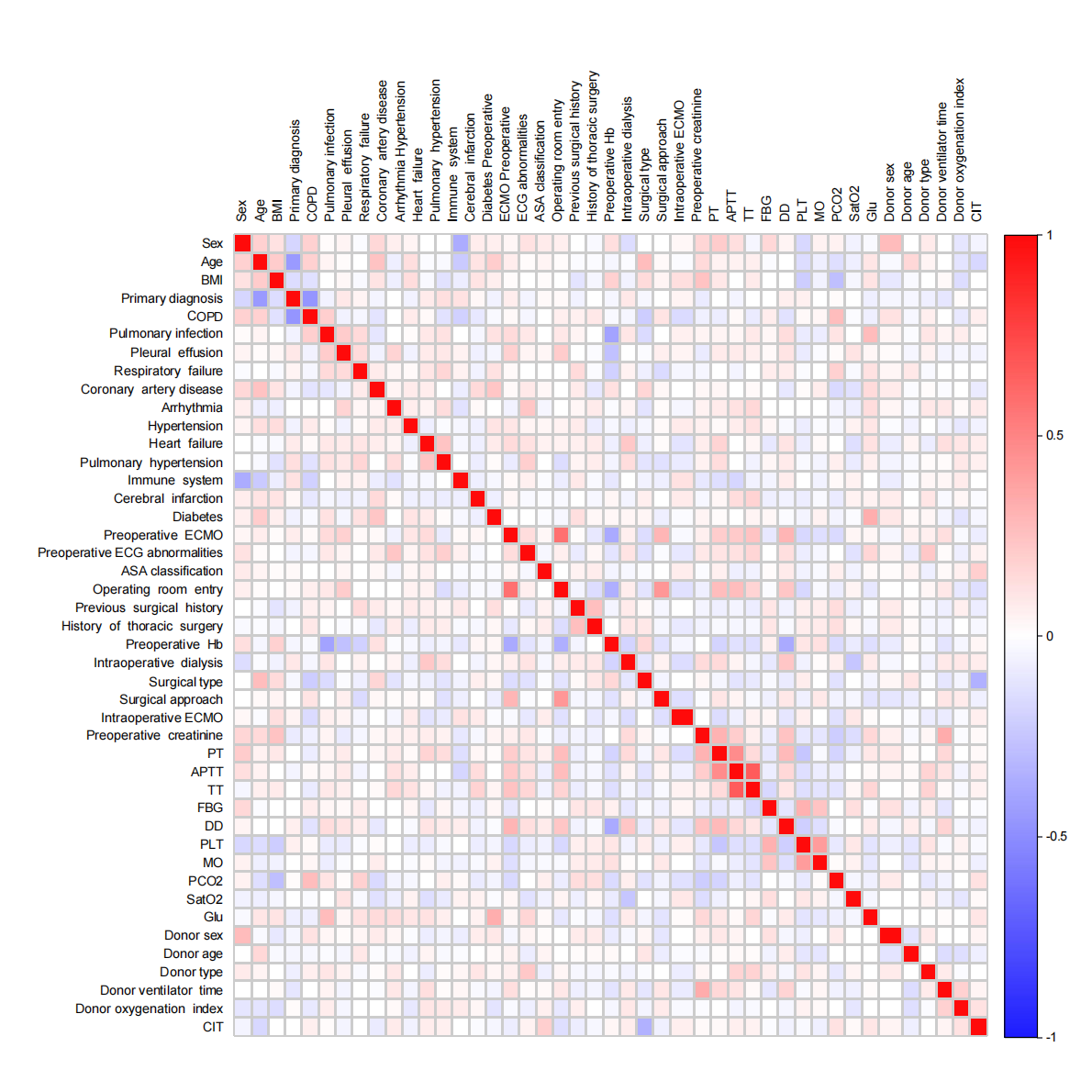


**sFigure 5.** Forest plots on OR values for three variable selection methods. (A) Results from the univariate and multivariate analysis with 6 predictors. (B) Results from the LASSO regression with 7 predictors. (C) Results from the RF-RFE combined with backward stepwise selection with 14 predictors. Abbreviations in this figure are the same as those described previously.
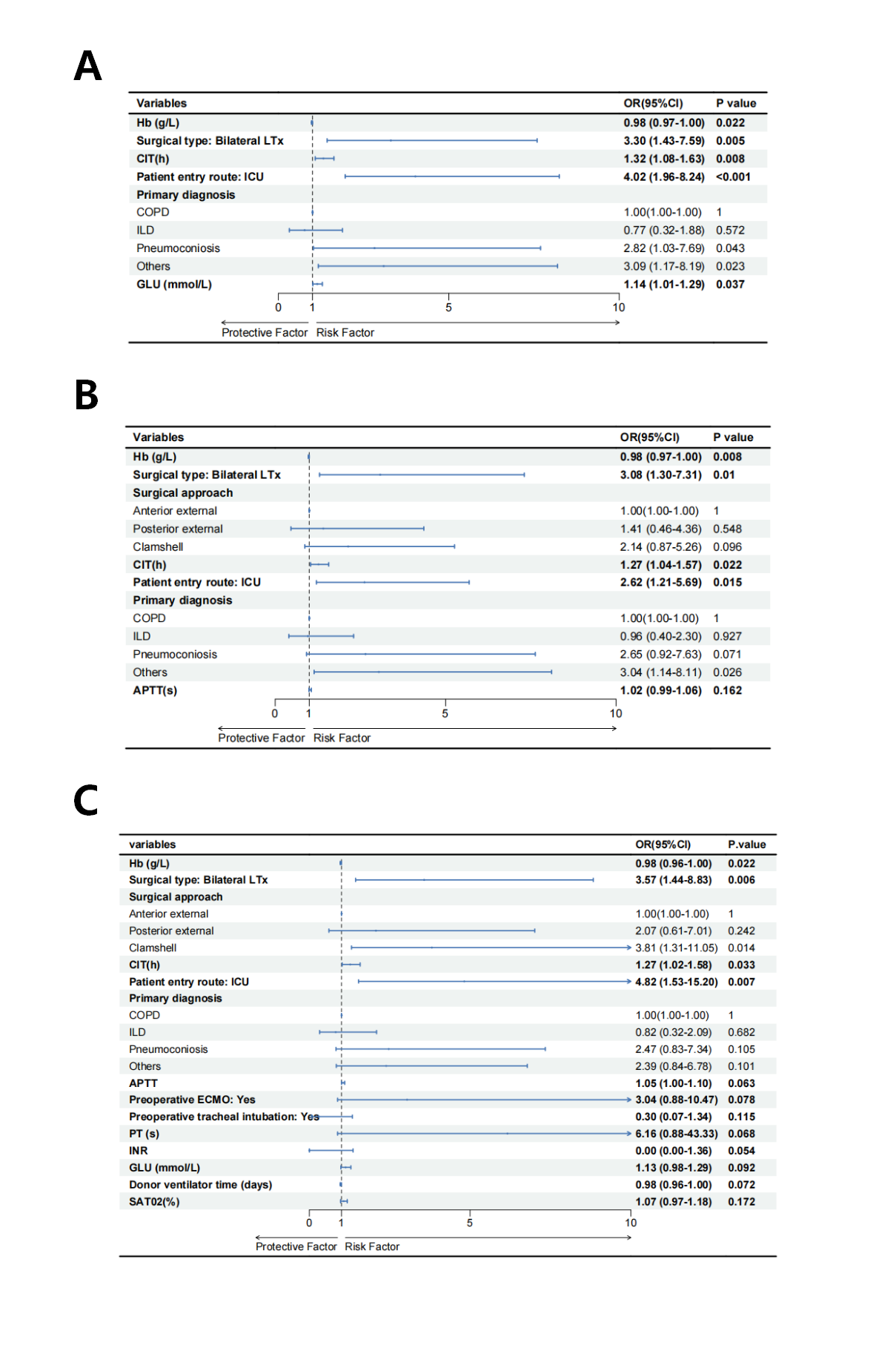


**sFigure 6.** Variable selection based on RF-RFE model with 10-fold cross-validation in the training cohort. (A) The RF-RFE model for feature variable selection had the highest accuracy when it included 29 predictive factors. (B) Relative importance of 29 confirmed predictors was shown in the histogram pattern. Abbreviations in this figure are the same as those described previously.
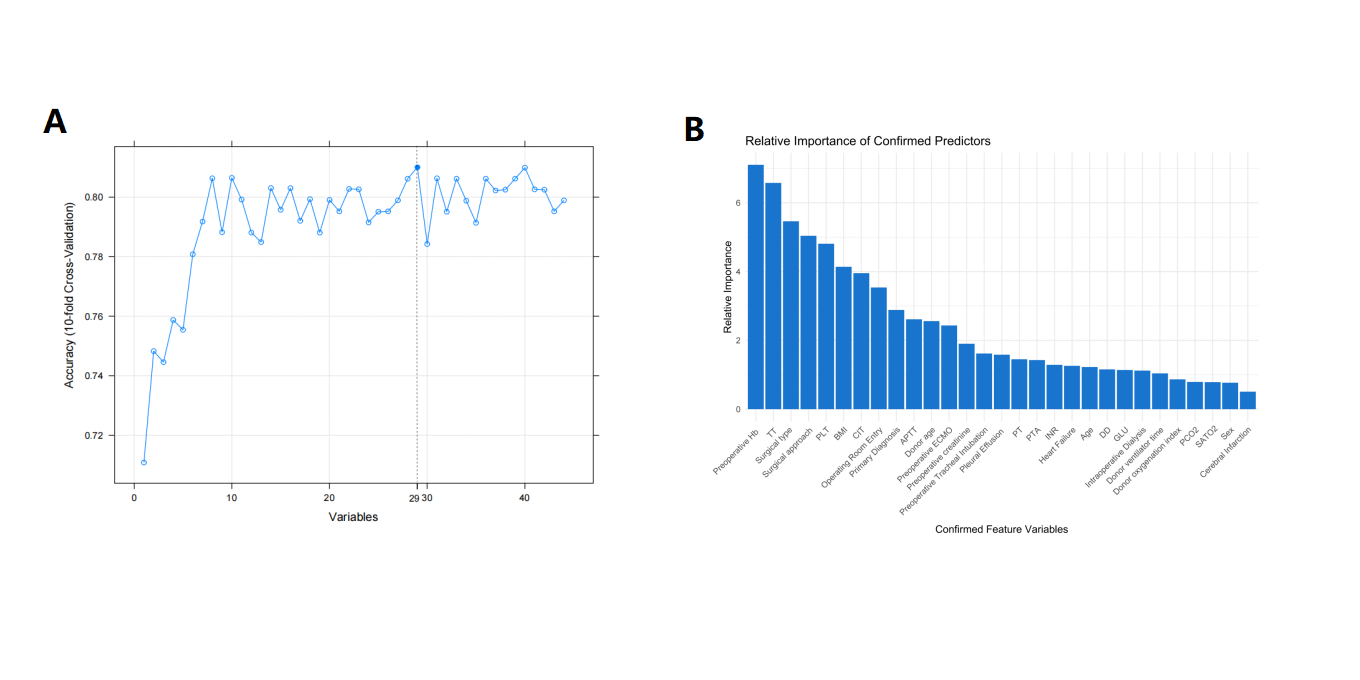


**sFigure 7.** Non-linear association between selected continuous variables and MT within patients from training cohort using univariate logistic model based on restricted cubic splines with 3 knots. (A) Hb; (B) APTT; (C) CIT; The break-point analysis based on the RCS model identified the apparent change points for determining the optimal thresholds for CIT as 8 hours.

**sFigure 8**. Decision curve analysis of prediction model for the training and test cohorts. (A) Decision curves for MT in the training cohort. (B) Decision curves for MT in the test cohort. Net benefits (blue lines) are shown as a function of the sequential probability thresholds for MT. The green and red lines indicate the hypothetical conditions when no or all patients have MT, respectively. Across a wide range of decision thresholds, the model showed satisfactory clinical significance with consistently positive net benefits.


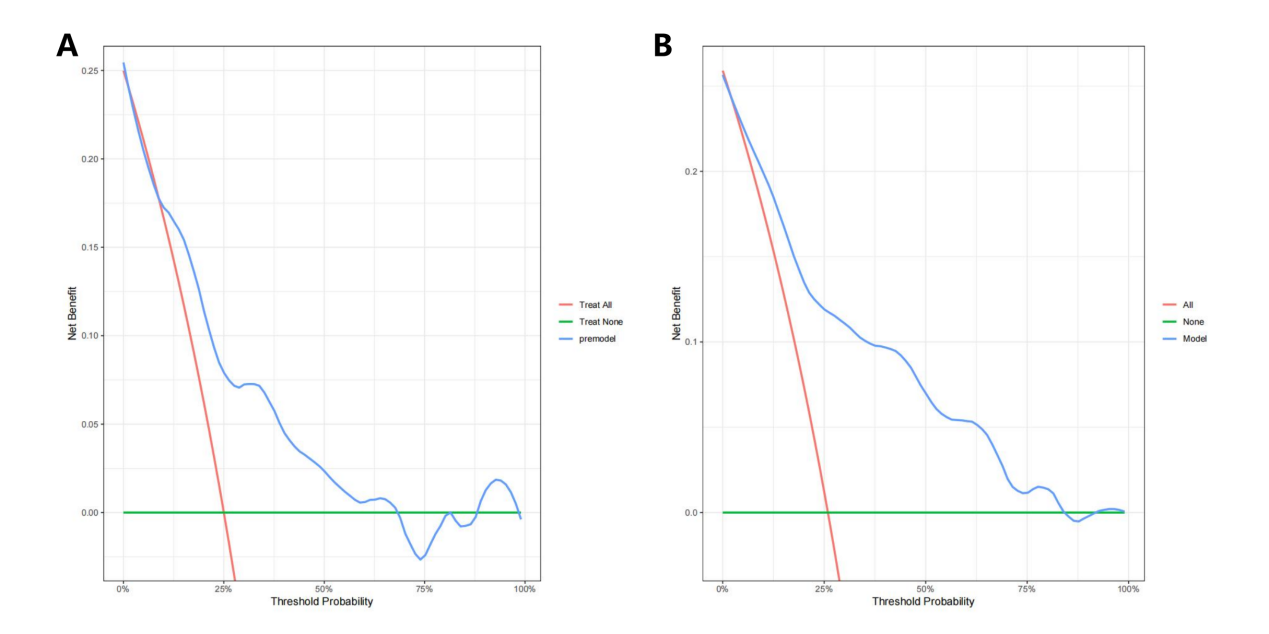

Supplement: Supplementary file 1 [file Table_1.docx]
