# Supplementary material for: Prediction of massive intraoperative blood transfusion requirement in lung transplantation
Source: Front Med (Lausanne). 2026 May 18;13:1786557. doi: 10.3389/fmed.2026.1786557 (PMC13222940; doi:10.3389/fmed.2026.1786557)
Supplement: Supplementary file 2 [file Table_2.docx]

sTable 1. Univariable and multivariable results based on the logistic model for identifying predictors for MT in the training cohort.

| **Variables** | | **Univariate Model** | | | **Multivariate Stepwise Model** | | |
| --- | --- | --- | --- | --- | --- | --- | --- |
|  | | **OR (95% CI)** | ***P*-value** | | **OR (95% CI)** | ***P*-value** | |
| **Baseline characteristics** |  | | |  |  |  |  |
| Age (years) | | 0.98 (0.96-1.00) | 0.118 | |  | |  |
| Sex (Female vs Male) | | 1.02 (0.48-2.15) | 0.964 | |  | |  |
| BMI (kg/m^2^) | | 0.92 (0.85-0.98) | 0.015 | |  | |  |
| **Comorbidities and medical history** | |  |  | |  | |  |
| COPD | | 1.23 (0.68-2.20) | 0.492 | |  | |  |
| Pulmonary infection | | 1.66 (0.96-2.86) | 0.069 | |  | |  |
| Pleural effusion | | 2.32 (1.12-4.80) | 0.023 | |  | |  |
| Respiratory failure | | 1.27 (0.68-2.37) | 0.452 | |  | |  |
| Coronary artery disease | | 0.78 (0.38-1.58) | 0.490 | |  | |  |
| Hypertension | | 1.12 (0.51-2.45) | 0.784 | |  | |  |
| Heart failure | | 1.82 (0.90-3.68) | 0.094 | |  | |  |
| Pulmonary hypertension | | 1.25 (0.73-2.16) | 0.417 | |  | |  |
| Cerebral infarction | | 1.15 (0.22-6.05) | 0.871 | |  | |  |
| Diabetes | | 1.40 (0.68-2.88) | 0.360 | |  | |  |
| **Surgery-related characteristics** | |  |  | |  | |  |
| Primary diagnosis | |  |  | |  | |  |
| COPD | | reference |  | |  | |  |
| ILD | | 0.66 (0.31-1.41) | 0.284 | | 0.77 (0.32-1.88) | | 0.572 |
| Pneumoconiosis | | 1.81 (0.76-4.35) | 0.182 | | 2.82 (1.03-7.69) | | 0.043 |
| Others | | 2.49 (1.06-5.83) | 0.036 | | 3.09 (1.17-8.19) | | 0.023 |
| ASA classification ( IV-V vs III) | | 1.45 (0.57-3.71) | 0.436 | |  | |  |
| Surgical type (Bilateral LTx vs Unilateral LTx) | | 5.94 (2.80-12.60) | <0.001 | | 3.30 (1.43-7.59) | | 0.005 |
| Surgical approach | |  |  | |  | |  |
| Anterior external | | reference |  | |  | |  |
| Posterior external | | 1.76 (0.72-4.31) | 0.218 | |  | |  |
| Clamshell | | 5.27 (2.49-11.14) | <0.001 | |  | |  |
| Previous surgical history | | 1.20 (0.67-2.15) | 0.544 | |  | |  |
| Preoperative ECMO | | 3.84 (2.06-7.15) | <0.001 | |  | |  |
| Preoperative tracheal intubation | | 3.26 (1.82-5.85) | <0.001 | |  | |  |
| Preoperative ECG abnormalities | |  |  | |  | |  |
| None | | reference |  | |  | |  |
| Supraventricular | | 0.90 (0.28-2.86) | 0.854 | |  | |  |
| Ventricular | | 1.30 (0.54-3.14) | 0.566 | |  | |  |
| Patient entry route (ICU vs Ward) | | 3.90 (2.21-6.86) | <0.001 | | 4.02 (1.96-8.24) | | <0.001 |
| Intraoperative Dialysis | | 2.86 (1.16-7.06,) | 0.023 | |  | |  |
| Intraoperative ECMO | |  |  | |  | |  |
| None | | reference |  | |  | |  |
| VV | | 1.37 (0.44-4.26) | 0.585 | |  | |  |
| VA | | 2.43 (0.70-8.48) | 0.164 | |  | |  |
| **Preoperative laboratory data** | |  |  | |  | |  |
| Hemoglobin(g/L) | | 0.97 (0.96-0.98) | <0.001 | | 0.98 (0.97-1.00) | | 0.022 |
| Platelets (10^9^/L) | | 1.00 (0.99-1.00) | 0.033 | |  | |  |
| Creatinine (µmol/L) | | 1.00 (0.99-1.01) | 0.884 | |  | |  |
| GLU (mmol/L) | | 1.09 (0.99-1.19) | 0.073 | | 1.14 (1.01-1.29) | | 0.037 |
| PT (s) | | 1.16 (0.97-1.38) | 0.109 | |  | |  |
| PTA (%) | | 0.98 (0.97-1.00) | 0.038 | |  | |  |
| INR | | 4.00 (0.69-23.06) | 0.121 | |  | |  |
| APTT (s) | | 1.05 (1.02-1.08) | 0.003 | |  | |  |
| TT (s) | | 1.01 (1.00-1.02) | 0.050 | |  | |  |
| FBG (g/L) | | 0.94 (0.75-1.18) | 0.600 | |  | |  |
| DD (ng/mL) | | 1.00 (1.00-1.00) | 0.068 | |  | |  |
| PCO_2_ (mmHg) | | 1.02 (1.00-1.04) | 0.039 | |  | |  |
| SATO_2_ (%) | | 1.15 (1.00-1.32) | 0.047 | |  | |  |
| **Donor-related characteristics** | |  |  | |  | |  |
| Donor sex (Male vs Female) | | 0.98 (0.47-2.08) | 0.964 | |  | |  |
| Donor age (years) | | 1.01 (0.98-1.03) | 0.619 | |  | |  |
| Donor type (DCD vs DBD) | | 0.81 (0.16-4.00) | 0.798 | |  | |  |
| Donor ventilator time(days) | | 0.99 (0.97-1.01) | 0.318 | |  | |  |
| Donor PaO_2_/FiO_2_ (mmHg) | | 1.00 (1.00-1.00) | 0.371 | |  | |  |
| CIT(h) | | 1.35 (1.13-1.61) | <0.001 | | 1.32 (1.08-1.63) | | 0.008 |

Abbreviations: LTx, lung transplantation; BMI, body mass index; COPD, chronic obstructive pulmonary disease; ILD, interstitial lung disease; ECMO, extracorporeal membrane oxygenation; ICU, intensive care unit; VV, veno-venous; VA, veno-arterial; GLU, glucose; PT, prothrombin time; PTA, prothrombin activity; INR, international normalized ratio; APTT, activated partial thromboplastin time; TT, thrombin time; FBG, fibrinogen; DD, D-dimer; PCO₂, partial pressure of carbon dioxide; SaO₂, arterial oxygen saturation; PaO₂/FiO₂, ratio of arterial oxygen tension to inspired oxygen fraction; CIT, cold ischemia time.
